# Baboon endogenous retrovirus (ERV) envelope pseudotyped lentiviral vectors outperform human ERV lentivectors for transduction of T, B, NK and HSPCs

**DOI:** 10.1038/s41434-025-00587-w

**Published:** 2026-01-19

**Authors:** Séverine Périan, Eva Castellano, Caroline Costa, Chiara Martinello, Anne Galy, Gisèle Froment, Rimas Orentas, Antonio Valeri, Els Verhoeyen

**Affiliations:** 1 CIRI – International Center for Infectiology Research, Inserm, U1111, Université Claude Bernard Lyon 1, CNRS, UMR5308, Ecole Normale Supérieure de Lyon, Université Lyon, Lyon, France; 2 Department of Translational Hematology, Research Institute Hospital 12 de Octubre (i + 12), Hematological Malignancies Clinical Research Unit H120-CNIO, CIBERONC, Madrid, Spain; 3 Université Côte d’Azur, INSERM, C3M, Nice, France; 4 ART-TG, US35, Inserm, Corbeil-Essonnes, France; 5 Lentigen Technology, Inc., a Miltenyi Biotec Company, Gaithersburg, MD USA

**Keywords:** Genetic vectors, Haematopoiesis, Targeted gene repair

## Abstract

Previously, we confirmed that BaEV-LVs outperformed VSV-G-LVs for gene delivery or correction of human T cells, B cells, NK cells and HSPCs correlating with high expression of its receptors, ASCT-1 and ASCT-2 on these cells. Since HERV-W gp uses the same entry receptors, we compared transduction efficiencies for BaEV-LVs and HERV-W-LVs in hematopoietic cells. HERV-W LV transduction was efficient but inferior to BaEV-LV in TCR-stimulated T cells (40% versus 80%) and this low efficiency was even more pronounced in IL-7/IL-15 pre-stimulated T cells. BaEV-LVs were significantly superior over HERV-W-LVs for the transduction of B cells and NK cells. High HERV-W-LV mediated transduction levels were achieved for pre-stimulated hCD34+ cells, which remained though lower than for the BaEV-LVs. Additionally, BaEV-LVs reached over 80% of transduction in severe combined immunodeficiency (SCID) repopulating cells (SRC) in 6/6 engrafted NBSGW mice. HERV-W-LVs reached this transduction level in 1/5 mice, while 3/5 engrafted NBSGW mice reached significantly lower transduction levels (20–50%). For both vectors the transduction levels were equivalent in the lymphoid and myeloid lineages in all hematopoietic tissues, suggesting transduction of immature HSPCs. Summarizing, BaEV-LVs outperformed HERV-W-LVs for transduction of important gene therapy target cells such as NK, B, T cells and CD34+ HSPCs.

## Introduction

Human endogenous retroviral sequences (HERVs), comprise a significant portion of the human genome (about 8%) [1, 2]. However, the majority of these HERVs are mutated or abrogated and do not encode anymore for functional proteins and even less likely for replication competent viruses. Some of these HERV sequences though still encode for complete viral proteins such as envelope glycoproteins (env gps), which are expressed at high levels in specific tissues. This is the case for the envelope gps of HERV-E, -R and -W virus, expressed naturally in the placental syncytia trophoblasts and are responsible for cell-cell fusion between neighboring cells, resulting in the formation of syncytia [3, 4]. HERV-W (syncytin-1) env gp in particular, plays a role in placenta formation [5–7].

Phylogenetic analysis of the HERV-W env gp suggests that it is related to the env gps of another group of retroviruses that include the feline endogenous retrovirus, RD114, the baboon endogenous retrovirus, BaEV, type D primate and simian retroviruses, which all use the human sodium-dependent Alanine, Serine, Cysteine transporter type 2 (hASCT2, gene name SLC1A5) as their common cell receptor [3, 8–12]. Specifically, BaEV gp can use hASCT1 as well as hASCT2 [10], whereas RD114 envelope gp only uses hASCT2 [8]. HERV-W env gps were shown to recognize hASCT-1 as well hASCT-2, similar to BaEV gps [11].

Importantly, both ASCT-1 and ASCT-2 are present on multiple tissues including hematopoietic cell such as T, B, NK cells and hematopoietic stem cells [13, 14]. For gene modification of these gene therapy targets in the resting state (as we find them in the blood), classical VSV-G pseudotyped LVs were not efficient because the VSV-G entry receptor, the low density lipid receptor (LDL-R), was poorly expressed on their surface [15, 16]. TCR- or cytokine stimulation for T-cells and cytokine pre-stimulation for HSPCs, inducing LDL-R expression, were required to obtain efficient gene transfer with VSVG LVs [17, 18]. These stimulations compromise, however, T cell phenotype and the ‘stem cell’ characteristics of HSCs [19]. For other targets such as NK and B cells even more hurdles need to be overcome to obtain high VSVG LV transduction since strong cytokine- or BCR-stimulation allowed only very poor transduction into these immune cells [20–22]. To overcome these short-comings, RD114 LV and BaEV LV pseudotypes were engineered by modifying their cytoplasmic tail [23, 24]. The RD114-LVs and BaEV-LVs achieved high-level transduction of cytokine pre-stimulated HSCs [23–26] with BAEV-LVs outperforming RD114-LVs explained by entry of BaEV env gp through an additional amino acid transporter (ASCT-1). Even more impressive, these BaEV-LVs permitted efficient gene delivery in HSPCs as compared to VSVG-LVs in the absence of cytokine stimulation thereby conserving their stem cell potential [24]. Additionally, it was confirmed that BaEV-LVs outperformed VSV-G-LVs for gene modification or correction of human healthy T cells, thymocytes, B cells and NK cells [18, 20–22, 27–30]. High-level transduction of all these targets by BaEV-LVs was correlated with high expression of both ASCT-1 and ASCT-2 receptors. Interestingly, several studies have shown that similar to BaEV env gp, HERV-W env gp can also use both hASCT-1 and hASCT-2 as entry receptors [3, 10–12].

Therefore, we generated lentiviral vectors pseudotyped with different modified HERV-W gps and identified the candidate that resulted in high titer HERV-W LVs. This HERV-W-LV was then compared with BaEV-LV for its capacity to genetically modify T cells, B cells, NK cells and HSPCs.

## Material and methods

### Plasmids

#### Envelope construction

**Fig. 1:**
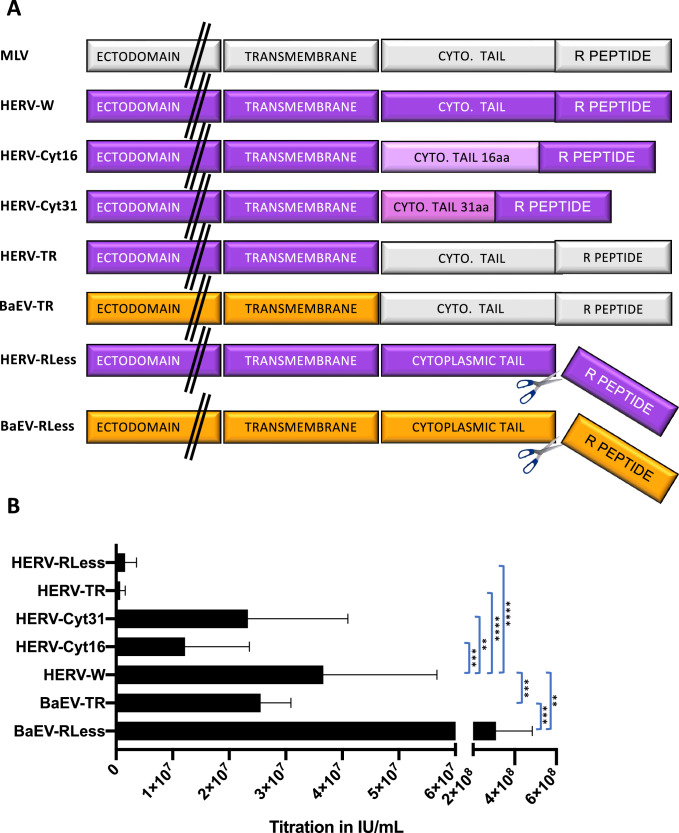
Unmodified HERV-W envelope gp allows efficient pseudotyping of lentiviral vectors. **A** Schematic representation of the different WT or cytoplasmic domain-modified viral envelope glycoproteins (gp) for pseudotyping of LVs. MLV, murine leukemia virus; HERV-W, human endogenous retrovirus V-W, HERV-Cyt16, HERV- Cyt31: cytoplasmic tail deletion mutants, HERVTR, HERV gp mutant carrying the cytoplasmic tail and R-peptide of the MLV gp; HERVRLess, HERV gp mutant lacking the R-peptide, BaEV baboon endogenous retrovirus gp, BaEVTR, BaEV gp carrying the cytoplasmic tail and R-peptide of the MLV gp, BaEVRless is a BaEV gp mutant lacking the R-peptide. **B** Titer of the different vector pseudotypes carrying a GFP reporter gene determined on 293 T cells by serial vector dilutions. Titer was analysed by FACS at day 3 post-transduction for GFP expression (IU/ml; mean ± SD; *n* = 6; two-way Anova, **p < 0.01, ***p < 0.001,****p < 0.0001).

By PCR-amplification the cytoplasmic tail of HERV-W wt was replaced by the one of MLV-A resulting in HERV R+ gp. The Herv R-gp was constructed by deletion of the R-peptide sequence from HERV-W (Fig. 1A). Truncated versions of the HERV-W envelope were generated by introduction of a stop codon after amino acid 485 leading to a cytoplasmic tail deleted for 16 aa (HERV-W-cyt16) or of a stop codon after amino acid 500 was introduced (Herv-cyt31) [11]. BaEVRless lacks the R-peptide, while the BaEVTR has it cytoplasmic tail exchanged for the one of MLV-A gp as described in Girard-Gagnepain et al. [24]

All chimeric envelope gps were expressed in the phCMV-G expression plasmid [19].

#### Production and titration of LVs

Self-inactivating HIV-1-derived vectors encoding green fluorescent protein (GFP) under the control of a spleen focus forming virus (SFFV) promoter were generated by transfection of 293 T cells as previously described [24]. Transfection was performed using the classical calcium phosphate method (solution A: 280 mM NaCl, 10 mM KCl, 1.5 mM Na_2_HPO_4_, 12 mM D-glucose, 20 mM Hepes, adjust to 7.2 with NaOH and solution B: 2 mM CaCl_2_). For display of the different BaEV and HERV-W gps, 7 µg of each envelope plasmid was transfected together with a Gag-Pol packaging plasmid (PACS) and a plasmid encoding a self-inactivating lentiviral vector (LV) expressing GFP under the control of the SFFV ([SIN]-HIVSFFVGFP). Viral supernatants were harvested 48 h after transfection and filtered over a 45 μm filter. The vectors were concentrated at low speed by overnight centrifugation of the viral supernatants at 3000 *g* at 4 °C. Titering of the LVs was performed on 293 T cells using serial dilutions.

#### Isolation of human blood cells

##### CD34^+^ cells isolation

Umbilical cord blood (CB) samples from full-term pregnancies (provided by EFS, Besançon, France) were collected after informed consent of donors and approval was obtained by the ethics committees of the hospitals according to the Helsinki Declaration. Low-density cells were separated by Ficoll gradient (Sigma-Aldrich, St Louis, MO, USA). CD34^+^ purification was performed by positive magnetic cell separation using the Automacs pro-separator (Miltenyi Biotec, Bergisch Gladbach, Germany) after staining of the cells with the human CD34^+^ MicroBead Kit (Miltenyi Biotec, cat #130-046-702,). Purity of the selected CD34^+^ cell fraction was evaluated by FACS analysis (Miltenyi Biotec MACSquant VYB) with APC-conjugated anti-CD34 antibody (Miltenyi Biotec, cat #130-113-176,). Cells were frozen in FCS/10% DMSO at −80 °C for later use.

##### Isolation and expansion of peripheral blood NK cells

Peripheral blood samples were obtained from healthy donors after informed consent and approval was obtained by the ethical committee of the hospital according to the Helsinki declaration. Hospital ethical committee approved-protocols. Peripheral blood mononuclear cells (PBMCs) were isolated by ficoll density gradient centrifugation and co-cultured with irradiated K526-mb21-41BBL feeder cells (CSTX002; kindly provided by Dean A. Lee, MD, PhD) in RPMI medium supplemented with 10% human AB serum (Sigma–Aldrich, St. Louis, MO, USA) and 100 U/ml human IL-2 (Miltenyi Biotec, Bergisch Gladbach, Germany) during 6 days for NK activation and expansion. Activated NK cells were then isolated by immunomagnetic depletion using an NK Cell Isolation Kit (Miltenyi Biotec) following the manufacturer’s protocol and maintained with continuous human AB serum and IL-2 stimulation. Purity of the culture was checked by flow cytometry after CD3-PECy7, CD16 APC-Cy7 and CD56-APC antibody staining (Biolegend, San Diego, CA, USA).

### T and B cell isolation

Peripheral blood samples were obtained from healthy donors after informed consent and approval was obtained by the ethical committee of the hospital according to the Helsinki declaration. PBMCs were isolated using Ficoll gradient (Sigma-Aldrich, St Louis, MO, USA). CD19^+^ B cells and CD3^+^ T cells were purified by positive selection using the human CD19 Microbeads (cat #130-050-301, Miltenyi Biotec) for CD19^+^ B cells and the human Pan T cells isolation kit (Miltenyi Biotec) for the CD3^+^ T cells following manufacturer’s instructions followed by separation through the Automacs pro-separator (Miltenyi Biotec). Purity of isolated B was monitored using anti-hCD19-APC antibodies (cat #130-113-647, Miltenyi Biotec), and was analyzed by flow cytometry (MACSQuant VYB, Milteny Biotec).

### Transduction of primary blood cells

#### Transduction of human CD34+ cells

Human CD34^+^-cells were incubated for 14 h or 18–24 h (as indicated) in 24-well plates in serum-free CellGro medium (Sartorius CellGenix GmbH, Freiburg, Germany) supplemented with human recombinant cytokines purchased from Miltenyi Biotec (Germany): SCF (100 ng/ml; # 130-096-692), TPO, (20 ng/ml; # 130-096-692), Flt3-L, (100 ng/ml; 130-096-474). Transductions were performed on retronectin coated plates according to manufacturer’s instructions (Takara Bio, Mountain View, CA, USA). 5 × 10^4^ prestimulated CD34^+^-cells per well were transduced in 48-well plates with concentrated LVs at indicated MOIs in serum-free medium. Cells were replenished with cytokines every 3 days. Three and 6 days after transduction, the % of GFP^+^-cells was determined by FACS.

### NK cell lentiviral transduction

Two days after purification, isolated NK cells were seeded at a confluency of 1.33 × 10^6^ cells/ml on culture plates previously coated with 2 μg/cm^2^ Retronectin (Takara Bio). Serum AB concentration in RPMI medium was reduced to 2% during transduction. Lentiviral vectors were added at the indicated multiplicity of infection (MOI) and cells were spinoculated for 1 h at 1000 *g* and 30 °C. Twenty-four hours after transduction, NK cells were washed and cultured in RPMI medium supplemented with 10% human AB serum and 100 U/ml IL-2. Fresh medium was added to the culture every 2–3 days. Cells were maintained in culture during 10 days.

Transduction efficiency was analyzed at different time points (day 5 and day 10) after transduction by flow cytometry.

### T cell transduction

Peripheral blood (PB) T lymphocytes were prestimulated for 3 days with T cell human hCD3/hCD28 TransAct beads (Miltenyi Biotec; #130-111-160,) supplemented with IL-2 (100 ng/ml) (Miltenyi Biotec; #130-097-743,) in RPMI or were prestimulated with recombinant 10 ng/mL human rIL-7 (rhIL-7; Miltenyi Biotec, #130-093-937) and 10 ng/ml rhIL-15 (Miltenyi Biotec 130-093-955,) for 3 days.

Next, 5E4 PB T cells upon TCR or cytokine stimulation were seeded in 48-well plates coated with RetroNectin® (Takara; #T100B; 12 µg/ml PBS according to manufacturer’s recommendations) and transduced with LVs at the indicated multiplicity of infection (MOI). Cell cultures were replenished with cytokines every 3 days. Three and 6 days after transduction, the percentage of GFP+ cells was determined by fluorescence-activated cell sorting (FACS).

### B cell transduction

B cells were stimulated for 24 h with 200 ng/ml Pansorbin A (Staph. Protein A (SpA; Sigma; #507858-1GM) and 100 ng/ml IL-2 (Miltenyi Biotec, #130-097-743,). 5E4 B cells were seeded in 48-well-plates coated with RetroNectin® (Takara Bio; cat #T100B; 12 µg/ml). and transduced with LVs at the indicated multiplicity of infection (MOI).

### Viability

Viability of T, B and CD34^+^ cells upon nanoblade incubation was determined using Annexin V/ propidium iodide staining and was then analyzed by FACS (MACSQuant VYB, Milteny Biotec).

### Conditioning and reconstitution of NBSGW mice with hCD34+ cells

NOD.Cg-*Kit*^*W-41J*^ *Tyr* ^+^ *Prkdc*^*scid*^ *Il2rg*^*tm1Wjl*^/ThomJ (NBSGW) mice used were housed in our animal facility (PBES-Lyon, France). Experiments were performed in accordance with the EU guidelines upon approval of the protocols by the local ethical committee (Autorization agreement N° C2EA -15: CECCAPP, Lyon, France). Detailed humanization protocol was described elsewhere [31]. Briefly, 3–5 week old female or male NBSGW mice were conditioned with Busulfex (20 mg/kg); 36 h later 7E4 CD34+ cells (transduced or not with LVs) were injected intravenously into suborbital eye vein. Follow-up of the humanization level was determined every 2 weeks in the blood from 6 weeks of engraftment. Staining for murine CD45 and human CD45 cells was performed to calculate the human immune reconstitution = % hCD45 cells/(%hCD45 cells+ %mCD45 cells). The mCD45 and hCD45 cells were monitored using anti-hCD45-APC and anti-mCD45-VioBlue antibodies (Miltenyi Biotec, cat # respectively 130-110-633/130-110-664,) and were analyzed by flow cytometry (MACSQuant VYB, Milteny Biotec).

### Phenotyping of human blood cells in reconstituted NBSGW mice

For the detection of transduced engrafted human cells in NBSGW mice, flow cytometry analysis was performed using APC-conjugated anti-hCD45 antibody for the detection of total GFP+ human cells engrafted in the bone marrow, thymus, peripheral blood and spleen. Staining of immune cells in the blood and the spleen was performed using antibodies from Miltenyi Biotec: anti-hCD45-VioBlue (#130-110-637) combined with anti-CD14-PE (#130-110-577), -CD56-PECy7 (#130-113-313), -CD4-APC (#130-113-222) and -CD8-PECy7 (#130-110-680). Staining of progenitors and early B-cell population in the BM is performed using anti-hCD34-APC (#130-113-176), anti-hCD19-PECy7 (#130-113-647), anti-CD10-APC (#130-093-450) from Miltenyi Biotec. Staining of thymocytes with anti-hCD3-PE (#130-116-560), anti-hCD8-PECy7 (#130-110-680) and anti-hCD4-APC (#130-113-222) is performed to screen for thymic subpopulations.

### Quantification and statistical analysis

Statistical analysis was conducted using Microsoft Excel 2013 and Prism software v6.0 (GraphPad Software, La Jolla, CA, USA). Results are indicated as means ± SD (standard deviation) in the figure legends unless otherwise indicated. For statistical testing of significance a student’s *t* test or ONE way ANOVA was used followed by Tukey range test to assess the significance among pairs of conditions, the test were justified by checking if there was a normal distribution or not; p-values and number of biological repeats are indicated in the figure legends. A *p* < 0.05 was considered to indicate statistical significance. For animal experiments, we used the software GPower 3.1 to reduce to the minimum the number of animals per experiment required to obtain statistical relevance. We performed a t-test with bilateral analysis which resulted in 8 animals per group. For the animal experiments, we used blinding for group allocation since we cannot predict the level of human cell engraftment. We included only the tissues from animals which were sacrificed because they reached humane endpoint according to pre-established ethical criteria. All flow cytometry data shown are representative of at least *n* = 3 reproduced biological repeats and this is indicated in the figure legend.

## Results

### Efficient pseudotyping of lentiviral vectors with HERV-W gp

To compare lentiviral vectors (LV) pseudotyped with HERV-W to BAEV glycoprotein (gp) pseudotyped LVs, we generated and evaluated several mutants of HERV-W with the objective to obtain high titers for HERV-W LVs. As previously engineered for BaEV gp [24], we constructed a HERV-WTR gp variant, in which the cytoplasmic tail of HERV-W gp was exchanged for the one of murine leukemia virus (MLV) env gp (Fig. 1A). Additionally, as for BAEV gp we made a variant that lacks the R-peptide (HERV-WRless). Since we had also shown that in some cases the cytoplasmic tail of viral gps is too long to be compatible with efficient incorporation into the lentiviral membrane, we made two HERV-W gps deleted for their cytoplasmic tail and resulting in a residual cytoplasmic tail of 16 or 31 aa called HERV-Wcyt16 and -cyt31, respectively (Fig. 1A). In contrast to BaEVRless-LVs, which was previously shown to result in significantly higher titer as compared to the wt BaEV gp pseudotype [24], the counterpart HERV-W gp without the R peptide (HERVRless) and the HERVTR showed significantly lower concentrated titers than the HERV-W LVs (Fig. 1B). The cytoplasmic tail deletion mutation of HERV-W cyt16 and cyt31 showed higher LV infectious titers than HERVRless and HERVTR, however, these were still significantly lower than HERV-W wt gp LVs, reaching up to 6 × 10^7^ IU/ml. This was around 2-fold lower than titers obtained for RD114TR- and gibbon ape leukemia virus (GALV)TR pseudotyped LVs and 10-fold lower than VSVG-LVs (Supplementary Fig. 1). Of note, the titers for BaEVRless LVs are higher here than previously reported by us [24] because we made a change in the production protocol for BaEVRless. The strong fusion of the producer cells due to expression of the BaEVRless protein was toxic to the producer cells. Therefore, we changed the culture medium 5 h instead of 16 h after transfection. This resulted in at least a 5- to 10-fold higher titer for the BaEVRLess LVs for the 5 h medium change as compared to the 16 h protocol. Using the same protocol did not improve the titers of the HERV-W LVs (*data not shown*). We choose the HERV-W wt gp that gave the highest titer of all the HERV-W mutant gps tested and called it hereafter HERV-W LV for further evaluation of its transduction efficiency  in primary blood cells in comparison to BaEVRless-LVs.

### BaEV-LVs are superior to HERV-W LVs for transduction of human T cells

**Fig. 2:**
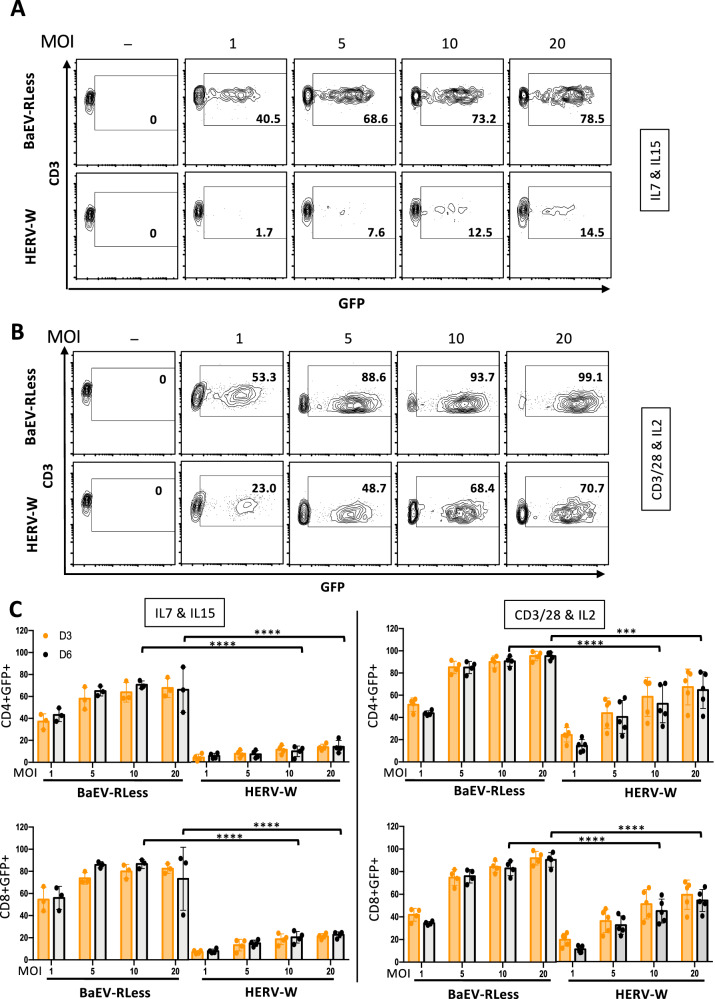
Comparison of BaEVRless-LVs and HERV-W-LVs for transduction of T cells. **A**, **C** Human T cells were pre-stimulated by IL-7 and IL-15 for 3 days and subsequently transduced with GFP encoding LVs pseudotyped with HERV-W or BaEVRless gps. The multiplicities of infections (MOI) are indicated. Representative FACS plots are shown in (**A**) and data are summarized in (**C**, left histograms). (mean ± SD; BaEVRless, *n* = 3; HERV-W, *n* = 4 two-way Anova,****p < 0.0001). **B**, **C** Human T cells were pre-stimulated by anti-CD3/anti-CD28 and IL-2 for 3 days and subsequently transduced with GFP encoding LVs pseudotyped with HERV-W or BaEVRless gps. The multiplicities of infection (MOI) are indicated. Representative FACS plots are shown in (**B**) and data are summarized in (**C**, right histograms). (mean ± SD; BaEVRless, *n* = 4; HERV-W, *n* = 5; two-way Anova, ***p < 0.001,****p < 0.0001).

Next, we evaluated transduction of BaEV-LVs and HERV-W LVs for T cells, that were pre-stimulated either by survival cytokines, IL-7 and IL-15 (Fig. 2A), or through the T cell receptor (TCR) by anti-CD3/anti-CD28 stimulation in the presence of IL-2 (Fig. 2B) for 3 days. Subsequent transduction with both LV pseudotypes showed an increasing transduction efficiency of IL-7/IL-15 stimulated T cells with increasing vector doses (MOI 1, 5, 10 and 20, Fig. 2A). However, the transduction with BaEV LVs reached 70–80% at an MOI of 10, while HERV-W LVs reached only 10%. At least a 4- to 5-fold lower transduction was detected in the CD4 + T cells and CD8 + T cells for HERV-W LVs as compared to BaEV LVs (Fig. 2C, left histograms). In contrast, TCR-stimulated T cells transduced with increasing vector doses (MOI 1, 5, 10 and 20, Fig. 2B) showed efficient transduction for the higher MOIs (10 and 20) used for the two different pseudotypes. However, HERV-W LV transduction efficiencies of CD4 + T cells and CD8 + T were significantly lower by 30% than those of BaEV LVs for the same vector doses (Fig. 2B and C, right histograms).

Concluding, though HERV-W LV transduction was efficient in TCR-stimulated T cells, its performance was inferior to the BaEV-LVs and this was even more pronounced for IL-7/IL-15 pre-stimulated T cells.

### BaEV-LVs are superior over HERV-W LVs for transduction of human B cells, NK cells and CD34 + HSPCs

Since BaEV-LVs previously were shown to transduce efficiently human B cells, NK cells and CD34+ cells [20–22, 24, 32], we wanted to evaluate the HERV-W LVs for transduction of the same gene therapy target cells.

**Fig. 3:**
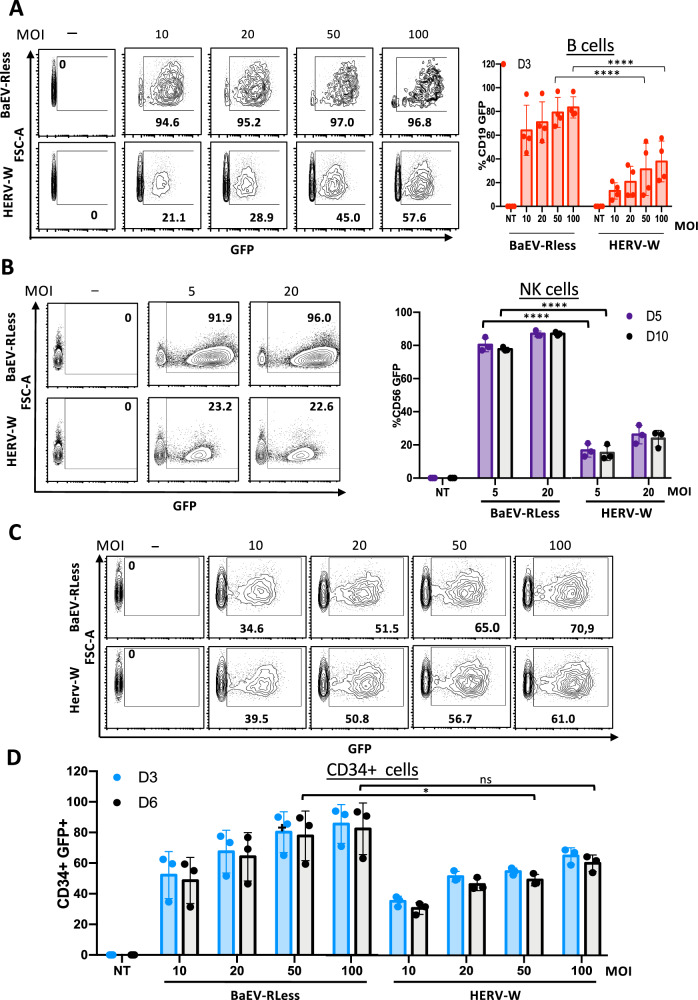
BaEVRless LVs are superior over HERV-W LVs for transduction of human B cells, NK cells and stem and progenitor CD34+ cells. **A** Fresh B cells isolated from PBMCs were pre-stimulated with Pansorbin A and IL-2 for 24 h and then transduced on retronectin coated plates with GFP encoding LVs pseudotyped with HERV-W or BaEVRless gps at indicated MOI. Representative FACS plots are shown for day 3 post-transduction and data are summarized for day 3 (**A**) (mean ± SD; BaEVRless, *n* = 4; HERV-W, *n* = 4; two-way Anova, ****p < 0.0001). **B** NK cells freshly isolated from PBMCs were expanded and continuously stimulated with AB serum and IL-2, before transduction. Then the NK cells were transduced on retronectin coated plates with GFP encoding LVs pseudotyped with HERV-W gp or BaEVRless gp at indicated MOIs. Representative FACS plots are shown for day 5 post-transduction and data are summarized for day 5 and day 10 in (**B**). (mean ± SD; BaEVRless, *n* = 3; HERV-W, *n* = 3; two-way Anova, ****p < 0.0001). **C** Human CD34+ cells from cord blood were prestimulated with TPO, SCF and Flk-3 ligand overnight and then transduced on retronectin coated plates with GFP encoding LVs pseudotyped with HERV-W gp or BaEVRless gp at indicated MOIs. Representative FACS plots are shown for day 6 post-transduction and data are summarized for day 3 and 6 post-transduction in (**D**). (mean ± SD; BaEVRless, *n* = 3; HERV-W, *n* = 3; two-way Anova, *p < 0.05, ns not significant).

As first targets, human B cells were activated through the BCR by Pansorbin and IL-2 overnight and transduced at different MOIs indicated (Fig. 3A). BaEV-LVs achieved for an MOI of 10 on average more than 60% transduction of B-cells, while HERV-W LVs achieved maximum 40% B-cell transduction at a 10-fold higher vector doses (MOI 100; Fig. 3A). As second target NK cells were generated by co-culture of PBMCs with irradiated K526-mb21-41BBL feeder cells for 6 days to activate and expand them. Subsequently, NK cells were isolated by positive selection and transduced at different MOIs with the two vectors as indicated (Fig. 3B). Though, BaEV-LVs reached already up to 90% transduction of NK cells at MOI of 20, HERV-W LVs did not exceed 25% at this vector doses (MOI 20; Fig. 3B).

Finally, as target cells, CD34+ cells were isolated by positive selection from cord blood and pre-stimulated overnight with a cocktail of early acting cytokines (TPO/SCF/Flk-3) before transduction with the two LV pseudotypes at indicated MOIs (Fig. 3C). Compared to the other target cells evaluated, HERV-W LVs achieved high levels of transduction of CD34+ cells (60%), however, high vector doses (MOI 100) were required to achieve this high level, while BaEV-LVs achieved this transduction level at an MOI ranging between 10 and 20 (Fig. 3C and D).

This means that as for T cells HERV-W LVs were inferior to BaEV-LVs for the transduction of B cells and NK cells. Higher HERV-W LV transduction levels were achieved for hCD34+ cells than for T cells, B cells and NK cells but nevertheless remained inferior to the performance of BaEV-LVs.

### HERV-W LV and BaEV LV engineered LVs transduce hHSPCs capable of long-term NBSGW mice reconstitution

The most frequently used mouse model for CD34+ cell engraftment in the context of HSC-based gene therapy evaluation is the non-obese diabetic (NOD)/severe combined immunodeficiency (SCID), γc −/− mouse model (called NSG [33]). However, CD34+ cell humanized NSG mice poorly support the development of shuman myeloid and erythroid lineages. Importantly, c-Kit receptor-mutant mice on the NSG background support unprecedented levels of human engraftment including myeloid-erythroid differentiation [34, 35]. One of these mice models, the NBSGW mice, supports an improved development of an innate immune system as compared to NSG mice. Therefore, these mice were selected to evaluate the long-term repopulation capacity of BaEV LV versus HERV-W LV transduced CD34+ HSPCs and their differentiation in as well lymphoid and myeloid lineages in vivo [36].

**Fig. 4:**
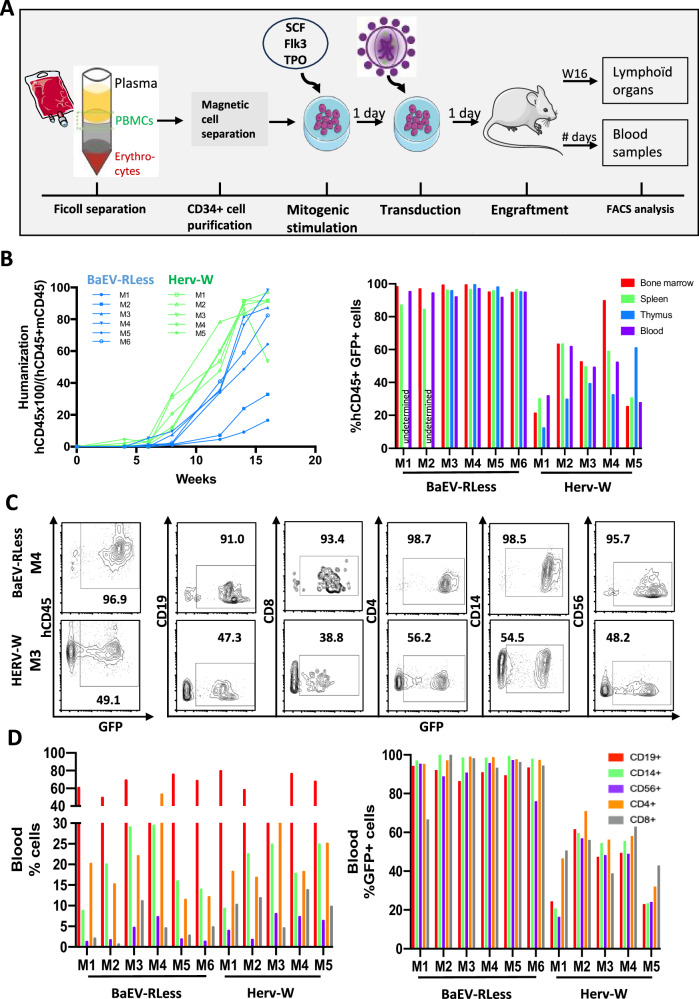
Humanization of immunodeficient NBSGW mice with HERV-W-LV or BaEV-LV transduced CD34+ cells confirmed persistence of modified cells upon differentiation into blood lineages. **A** Schematic representation of the experimental set-up; CD34+ cells are isolated from cord blood, subsequently stimulated with SCF/TPO/Flk3 overnight and then transduced with HERV-W LV or BaEVRless-LV at an MOI of 20. After 24 h of transduction CD34+ cells were injected into NBSGW mice (*n* = 6 for BaEVRless LVs; *n* = 5 for HERV-W LVs) to allow reconstitution of the mice with a human blood system for 16 weeks before sacrifice and FACS analysis of the different hematopoietic tissues. **B** Detection of humanization in the blood of the recipient mice reconstituted with BaEVRLess or HERV-W LV transduced CD34+ cells. **C** Percentage of transduced (GFP + ) per total hCD45+ cells in bone marrow, spleen, thymus and blood at mice sacrifice for BaEVRless LVs (M1 to M6) and HERV-W LVs (M1 to M5). ND thymus not detected. **D** Representative FACS plots for GFP+ cells in the blood for the different lymphocyte subpopulations (CD19 for B cells; CD4 and CD8 for T cells, CD14 for monocytes and CD56 for NK cells) for mice M4 in the BaEVRless LV group and the mice M3 in the HERV-W group. Distribution of different hematopoietic lineages in the blood of humanized NBSGW mice a sacrifice (left histogram); Data for the transduction of  different blood cell lineages (**D**, right histogram) are summarized for all the mice per vector group.

**Fig. 5:**
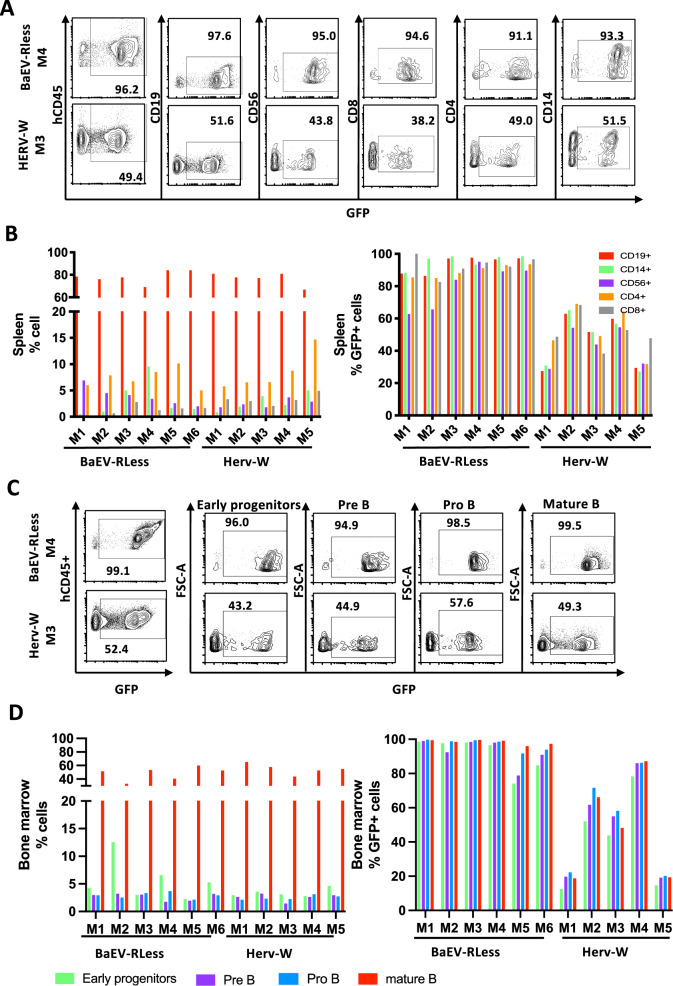
Humanization of NBSGW mice with HERV-W-LV or BaEV-LV transduced CD34+ cells confirmed persistence of transduced SCID repopulation cells. The experimental set-up for the humanization is outlined in Fig. 4A. **A** Representative FACS plots for GFP+ cells in the spleen for the different lymphocyte subpopulations (CD19 for immature and mature B cells; CD4 and CD8 for T cells, CD14 for monocytes and CD56 for NK cells) for mice M4 in the BaEVRless LV group and the mice M3 in the HERV-W group. **B** Distribution of different hematopoietic lineages in the spleen of humanized NBSGW mice a sacrifice (left histogram) and data from (**A**) are summarized for all the mice per vector group (right histogram). **C** Representative FACS plots for GFP+ cells in the BM for the different lymphocyte subpopulations (early progenitors (CD34 + CD19-CD10-), pro-B cells (CD34 + CD19-CD10 + ) and pre B cells (CD34l^ow^CD19 + CD10 + ) and CD19+ immature/mature B cells) for mice M4 in the BaEVRless LV group and the mice M3 in the HERV-W group. **D** Distribution of different hematopoietic lineages in the BM of humanized NBSGW mice a sacrifice (left histogram) and data from (**C**) are summarized for all the mice per vector group (right histogram).

In Fig. 4A, the experimental set-up is outlined: isolated CD34+ cells were pre-stimulated with a cytokine cocktail (SCF, TPO and Flk3) for 24 h followed by transduction at MOI 20 with the two vector pseudotypes for 36 h. Then they were injected into the blood stream of NBSGW mice, preconditioned with Busulfex. The mice were sacrificed 16 weeks post-engraftment for FACS analysis. A striking difference was detected in the humanization of the NBSGW mice reconstituted with BAEV LV or HERV-W LV transduced CD34+ cells. A faster humanization was revealed for the latter group (Fig. 4B). The % of human leukocytes (hCD45+cell) that expressed GFP is shown in Fig. 4C. High level hCD45+ cell transduction was detected for the BaEV LVs (>95%) which was equivalent in thymus, spleen, BM and blood for 6 NBSGW recipients. In contrast, the % of transduced hCD45+ cells in HERV-W LV injected mice varied between recipient mice and for each mouse between hematopietic tissues (Fig. 4C). In the blood at sacrifice, all mice (HERV and BaEV LV) showed as expected more B cells (CD19 + ) than T cells (CD4+ and CD8 + ). Additionally, the CD14+ monocyte levels were high in all the mice and natural killer cells were readily detected (CD56+ cells) as expected in humanized NBSGW mice (Fig. 4D). Further detailed immune cell phenotyping in the blood for the BaEV LV group showed that CD4+ and CD8 + T and B lymphocytes as well as monocytes and natural killer cells showed equivalent and high GFP levels (>80%; Fig. 4D). This transduction profile was confirmed for the 6 recipient mice. Although in the HERV-W LV group, each recipient mice showed GFP transduction with some exceptions to the same extent between the different blood lineages, an important variability in % of GFP was detected between the 5 individual engrafted animals. A similar GFP transduction and hematopoietic lineage profile as in the blood was seen for BaEV LV (M1 to M6) and HERV W LV (M1 to M5) conditions in the different lymphoid (CD4, CD8 T cells and B cells) and myeloid (CD14+ monocytes and CD56+ natural killer cells) lineages of the spleen (Fig. 5A and B).

Detailed phenotyping of the bone marrow, again confirmed the GFP transduction profile as found in the other hematopoietic tissues (Fig. 5C). The different subpopulations, early progenitors (CD34 + CD19-CD10-), pro-B cells (CD34 + CD19-CD10 + ) and pre B cells (CD34l^ow^CD19 + CD10 + ) and CD19+ immature/mature B cells, showed equivalent lineage distributions in the two mouse groups (Fig. 5D). Moreover, high GFP transduction levels (>80%) were found in the BM for the BAEV-LV recipients (Fig. 5C and D). This transduction profile was consistent for the 6 recipient mice. Although for the HERV-W LV group, each recipient mice showed GFP transduction to the same extent between the different BM subpopulations, an important difference in level of GFP expression was detected between the 5 engrafted animals ranging from 20 to 80% (Fig. 5D). Finally, the same transduction profile was confirmed in the thymus for BaEV LVs for four engrafted mice (M3 to M6) with equivalent % GFP + cells in the thymic subpopulations (CD4 SP, CD8CD4 DP, CD8SP). In 2 out of 6 mice no thymus was detected for this group. In contrast, in the HERV-W LV group, transduction levels within the thymic subpopulations were not equivalent (Supplementary Fig. 2).

Summarizing, BaEV LV transduced CD34+ cells showed a much better reproducible and high-level transduction of long-term SCID repopulating cells as compared to HERV-W LVs.

## Discussion

We have generated high titer vectors pseudotyped with the unmodified wt HERV-W envelope. The attempt to improve these titers by introducing cytoplasmic tail deletions or  a waps with the MLV envelope cytoplasmic tail did not improve HERV-W LV titers. Whereas HERV-W and BAEV gp pseudotyping would have been expected to deliver genes with similar efficacy considering that these envelope glycoproteins were reported to utilize the same entry receptors, the results showed that BaEV LVs outperformed HERV-W LVs for the transduction of mildly IL-7 stimulated T-cells, cytokine activated NK and B cells in the experimental conditions used. HERV-W LVs did show high level transduction of T cells when stimulated through the TCR but for same vector doses (IU/ml), BaEV LVs outcompeted them. At high vector doses HERV-W LVs did transduce cytokine pre-stimulated CD34+ HSPCs, which were able to reconstitute immunodeficient mice including the engraftment of different lymphoid and myeloid hematopoietic lineages. For an equivalent vector doses though, BaEV LVs reached over 80% of transduction in severe combined immunodeficiency (SCID) repopulating cells (SRC) in 6/6 engrafted NBSGW mice, while HERV-W LVs only reached this for 1/5 mice while 3/5 engrafted NBSGW mice only reached 50% SRC transduction or lower (20%). Summarizing, BaEV LVs outperformed HERV-W LVs for gene delivery into important gene therapy target cells (NK, B, T cells and CD34+ HSPCs). However, HERV-W LVs did allow higher transductions of B and NK cells as compared to VSVG-LV transduction as previously reported [17, 20, 21].

B-cells are currently also in the spotlights for cell therapy since they can be employed as very potent producers of therapeutic antibodies such as e.g. checkpoint inhibitors used for immunotherapies. The long-term antibody secretion from autologous B cells may proof to be a promising treatment of autoimmune diseases, cancer and infectious diseases [22, 37–39]. One study by Coquin et al. shows the efficient transduction of unstimulated human B cells using HERV-W LVs, which was not tested here. Of note, important technical differences exist between this work and the current study, which indicates that optimization of the use of HERV-W glycoproteins may be required to optimize their application in gene therapy (bioRxiv 816223; 10.1101/816223).

Especially T cells and CD34+ cells, which are valuable target cells for gene therapies were difficult to genetically modify, at least by VSVG-LVs unless these cells were strongly activated by cytokines and transduced at high vector doses [17]. The VSV-G LV pseudotype is routinely used for gene transfer into T cells for CAR T cell generation and into CD34 HPCs for correction of monogenetic diseases [40–49]. Meanwhile, competitor LV pseudotypes have emerged such as RD114 LVs and Gibbon Ape Leukemia Virus (GALV) enveloped LVs, measles virus gp pseudotyped LVs as also BAEV LVs, which were shown to be efficient LV pseudotypes for T cell and HSPC gene modification. What’s more, they were even performing significantly better than VSVG-LVs in the absence of cytokine stimulation of these targets [18, 23, 24, 50]. Here we showed that HERV-W LVs did allow significantly high transduction of TCR-stimulated T cells and HSPCs, however, they were outperformed by BaEV-LVs at lower vector doses (Fig. 2 and Fig. 3C). Of note, HERV-W LV transduced CD34+ HSPCs were able to engraft immunodeficient NBSGW and give rise to all myeloid and lymphoid lineages in the different hematopoietic tissues. Since transduction efficiencies were within one mice equivalent between the different cell types, we can conclude that SCID repopulating cells were transduced.

For transduction of the CD34+ cells at equivalent vector doses, the BaEV LVs transduced significantly more SRCs as compared to HERV-W LVs since the former showed for all the recipient mice over 80% transduction in all the hematopoietic cell lineages within each of the tissues (spleen, blood, bone marrow and thymus).

For B cells the situation is more dramatic since, even upon stimulation the receptor of VSVG for entry, the LDR-receptor, was hardly expressed and thus gene modification was very poor [17, 21, 51]. B cells turned out to be relatively resistant to lentiviral transduction, even though a number of different envelope pseudotypes have been tested [52, 53]. Fortunately, RD114, GALV and BaEV env gp based LVs achieved high level transduction of B cells as shown here and elsewhere [17, 21, 22, 51]. HERV-W LVs again transduced stimulated B cells quite efficiently (up to 40%) but only at high vector doses (MOI 50 to 100), while BaEV-LVs reached 80% of transduction at the same vector doses (Fig. 3A). BaEV-LVs transduced as well unstimulated as stimulated B cells at high efficiency [21]. LVs pseudotyped with modified glycoproteins from measles virus (MV) have been used for B cell transduction [54, 55]. However, MV-pseudotyped LVs do have some limitations, in particular they are produced at relatively low titers.

One major obstacle when using primary NK cells in immunotherapy is the lack of an efficient gene transfer method. Interestingly, two independent studies showed that this hurdle could be overcome by switching the VSV-G envelope glycoprotein (gp) at the surface of a LV for a baboon endogenous retroviral (BaEV) envelope gp [24]. These LVs ensured up to 80% genetic modification of activated NK cells [20, 32, 56–58] Another study also showed high-level CAR delivery into NK cells by employing an α-retroviral vector pseudotyped with RD114 envelope gp [59]. Similar as for B cells, HERV-W LVs performed quite poorly for transduction of NK cells when compared to RD114 and BaEV LV pseudotypes (Fig. 3B).

Importantly, the high BaEV-LV transduction efficiencies of NK, T, B and HSC have been contributed to the high-level expression of both hASCT1 and hASCT2 receptors [18, 20, 60]. Indeed RD114-LVs performed less efficient for transduction of HSCs since these LVs only recognize a single amino acid transporter (ASCT2) [8] and this was especially confirmed in resting CD34+ HSPCs [24]. Both HERV-W LVs and BAEV LVs can efficiently use hASCT2 as receptor and also the related transporter hASCT1 as an auxiliary receptor [10, 11], whereas RD114 and type D simian retroviruses cannot use ASCT1. This was shown via a side by side comparison for transduction of CHO cell lines exclusively expressing ASCT-1 or ASCT-2. Fusion capacity of the BaEV gp for both receptors was higher than for the HERV-W envelope gp and the entry through both receptors was shown to be effective for both envelopes. Nevertheless, BaEVRless showed 8-fold higher transduction through both ASCT-1 and -2 receptor as compared to HERV-W. More recently, the fact that HERV-W is recognizing both ASCT-1 and -2 receptors was challenged by Stafl et al. [61] They put forward exclusively ASCT-2 as the sole receptor for binding of HER-W env gp, its cellular entry and cell-to-cell fusion. They detected a heteromeric complementation between ASCT-1 and -2 molecules and this led to the false interpretation that ASCT-1 also was a HERV-W receptor. This might explain why BAEV-LVs outperform HERV-W LVs for transduction of all the primary hematopoietic cells tested here.

We have evaluated the expression of the BaEV and HERV-W receptors, ASCT-1 and ASCT-2 previously under the same conditions of T, NK and HSC stimulation used in the current manuscript and compared them to the receptor expression in their resting state.

Indeed, BaEV-LVs permitted efficient transduction of HSPCs cells upon stimulation with a single cytokine (e.g., TPO or SCF). Moreover, BaEV-LV binding and signaling through both aa transporters, ASCT-1 and ASCT-2, was shown sufficient to transduce unstimulated HSPCs with the objective to avoid their differentiation and loss of the HSC subpopulation. Importantly, both ASCT-1 and ASCT-2 mRNA levels are upregulated upon TPO/SCF/Flk-3 cytokine-stimulation [24], which might strongly increase the affinity of BaEV-LVs to HSPCs and explain higher transduction levels in these stimulated cells as compared to their resting counterparts. ASCT-1 was 2.5-fold upregulated while ASCT-2 was 6-fold upregulated in stimulated versus resting HSPCs.

Furter BaEV-LVs obtained 30% transduction in freshly-isolated human NK-cells (FI-NK) and 80% transduction in NK-cells obtained from the NK-cell Activation and Expansion System (NKAES). Expression of both BaEV receptors, ASCT1 and ASCT2, was detected in FI-NK and NKAES, with higher expression in NKAES and a 10-fold upregulation of both receptors versus unstimulated NKs [20] again confirming that BaEV LV transduction of NK cells is dependent on these receptors. In contrast, BaEV LVs performed poorly on these quiescent T cells. Importantly, mRNA levels of both BaEV receptors (ASCT-1 and ASCT-2) were upregulated upon IL-7 stimulation, and the upregulation was even more pronounced after TCR-activation of T cells (Fig. 1 in Bernadin et al. [18]). This coincided with higher transduction levels obtained in these pre-stimulated T cells compared with their resting counterparts. Upon stimulation ASCT-2 was 16-fold upregulated in TCR-stimulated T cells, while ASCT-1 was only 6-fold upregulated.

We therefore speculate that since HERV-W LVs perform less efficiently in activated T, NK, B cells and HSCs than their BaEV-LV counterparts that they will be even forming poorer when these cells are in the resting state, since receptor expression is significantly lower. In this context we might speculate that the affinity of HERV-W gp to both ASCT-1 and ASCT-2 is lower or as recently published that HERV-W gp only recognizes ASCT-2 [61]

In conclusion, BaEV and HERV-W LVs though they are believed to use the same entry receptors and both can be generated at higher titers, the BAEV-LVs allowed more efficient gene delivery in human T, B, NK cells and HSPCs than detected for HERV-W LVs. Moreover, BaEV-LVs can be utilized to obtain stable producer cell lines. Both characteristics are huge advantages for gene therapy applications.

## Supplementary information

Supplemental material

## Data Availability

Most of the data generated or analysed during this study can be found within the published article and its supplementary files. All further data supporting the conclusions of this article are available from the corresponding author upon request.
