# Supplementary material for: Baboon endogenous retrovirus (ERV) envelope pseudotyped lentiviral vectors outperform human ERV lentivectors for transduction of T, B, NK and HSPCs
Source: Gene Ther. 2026 Jan 19;33(2):144–55. doi: 10.1038/s41434-025-00587-w (PMC13056528; doi:10.1038/s41434-025-00587-w)
Supplement: Supplementary file 1 — Supplemental material [file 41434_2025_587_MOESM1_ESM.pdf]

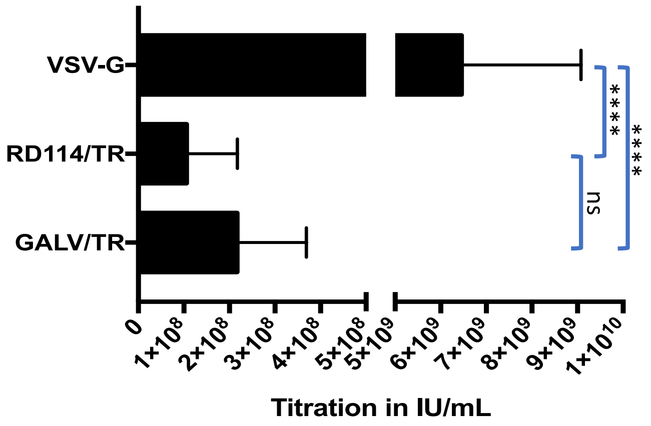

Supplementary Figure 1: Comparison of titers of concentrated vectors pseudotyped with different envelope gps.

Titer of the different vector pseudotypes carrying a GFP reporter gene determined on 293T cells by serial vector dilutions. Titer was analysed by FACS at day 3 post-transduction for GFP expression (IU/ml; mean±SD; n=6; two-way Anova,****p<0.0001, ns: not significant). VSV-G: vesicular stomatitis virus G protein; RD114/TR: cat endogenous retroviral envelope gp with its cytoplasmic tail switch for the one of MLV envelope gp. GALV/TR: gibbon ape leukemia virus envelope gp with with its cytoplasmic tail switch for the one of MLV envelope gp.

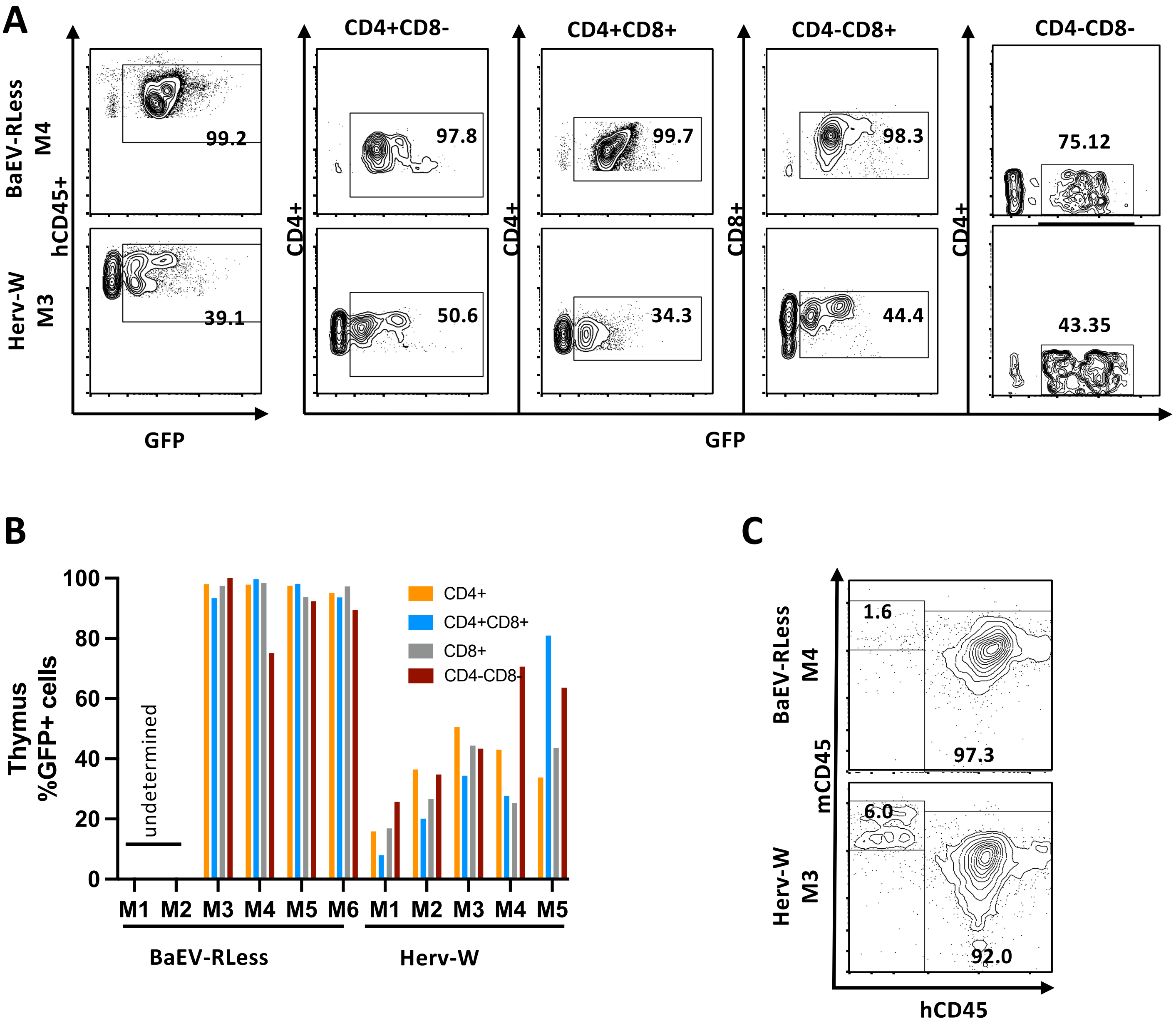

Supplementary Figure 2: Humanization of NBSGW mice with HERV-W-LV or BaEV-LV transduced CD34+ cells confirms persistence of modified cells in the thymus.
The experimental set-up for the humanization is outlined in Figure 4A.
(A) Representative FACS plots for GFP+ cells in the thymus for the different lymphocyte subpopulations (CD4+ CD8-CD3-for CD4 single positive thymocytes ; CD4+ and CD8+ for double positive thymocytes and and CD8+CD4- for CD8 single positive cells for mice M4 in the BaEVRless LV group and the mice M3 in the HERV-W group. (B) Data from A are shown for all the mice per vector group. ND = thymus was not detected. (C) Representative FACS plots for humanization of the thymus.
